# Developing a Novel Prosthetic Hand with Wireless Wearable Sensor Technology Based on User Perspectives: A Pilot Study

**DOI:** 10.3390/s24092765

**Published:** 2024-04-26

**Authors:** Yukiyo Shimizu, Takahiko Mori, Kenichi Yoshikawa, Daisuke Katane, Hiroyuki Torishima, Yuki Hara, Arito Yozu, Masashi Yamazaki, Yasushi Hada, Hirotaka Mutsuzaki

**Affiliations:** 1 Department of Rehabilitation Medicine, Institute of Medicine, University of Tsukuba, Tsukuba 305-8575, Japan; 2 Ibaraki Prefectural University of Health Sciences Hospital, Ami 300-0331, Japan; 3 Department of Electrical and Electronic Engineering, Shonan Institute of Technology, Fujisawa 251-8511, Japan; 4 Saitama Prosthetics and Orthotics Manufacturing Service Co., Ltd., Saitama 337-0051, Japan; 5 Department of Neurophysiology, National Center of Neurology and Psychiatry, Kodaira 187-8551, Japan; 6 Department of Precision Engineering, University of Tokyo, Bunkyo 113-8656, Japan; 7 Department of Orthopaedic Surgery, Institute of Medicine, University of Tsukuba, Tsukuba 305-8575, Japan; 8 Center for Medical Science, Ibaraki Prefectural University of Health Sciences, Ami 300-0331, Japan

**Keywords:** electric prosthetic hand, upper-limb deficiency, wireless wearable sensors, prosthetic hand user perspective, three-dimensional printer

## Abstract

Myoelectric hands are beneficial tools in the daily activities of people with upper-limb deficiencies. Because traditional myoelectric hands rely on detecting muscle activity in residual limbs, they are not suitable for individuals with short stumps or paralyzed limbs. Therefore, we developed a novel electric prosthetic hand that functions without myoelectricity, utilizing wearable wireless sensor technology for control. As a preliminary evaluation, our prototype hand with wireless button sensors was compared with a conventional myoelectric hand (Ottobock). Ten healthy therapists were enrolled in this study. The hands were fixed to their forearms, myoelectric hand muscle activity sensors were attached to the wrist extensor and flexor muscles, and wireless button sensors for the prostheses were attached to each user’s trunk. Clinical evaluations were performed using the Simple Test for Evaluating Hand Function and the Action Research Arm Test. The fatigue degree was evaluated using the modified Borg scale before and after the tests. While no statistically significant differences were observed between the two hands across the tests, the change in the Borg scale was notably smaller for our prosthetic hand (*p* = 0.045). Compared with the Ottobock hand, the proposed hand prosthesis has potential for widespread applications in people with upper-limb deficiencies.

## 1. Introduction

Prosthetic hands are indispensable tools for individuals with upper-limb deficiencies in activities of daily living (ADLs). The optimal use of prosthetic hands can significantly enhance the quality of life [1]. While various types of functional myoelectric hands are currently available, their acquisition and sustained use are impeded by several barriers [2,3,4,5,6], chiefly due to increased costs [7,8]. Individuals with upper-limb deficiencies often face challenges in procuring electrically powered prostheses without government assistance owing to their considerable costs; additionally, only a few healthcare systems allow the prescription of myoelectric hands [9,10,11,12]. In Japan, myoelectric prostheses are prescribed with government support only in extremely limited circumstances [11]. Funding structures responsible for prescribing prostheses vary internationally as well as regionally and depend on prosthetic users’ backgrounds, such as age and occupation. The abandonment of prostheses is another concern [2,4,5,6,13,14], which is often related to funding [12]. The weight of myoelectric prostheses is a contributing factor to their discontinued use [5,6,8,13]. Shoulder pain was prevalent among individuals with upper-limb deficiencies and associated with negative functional and quality-of-life outcomes [15]. Myoelectric hands are typically heavier than cosmetic or body-powered hands; therefore, they may impact the users’ necks and shoulders.

In addition, individuals with short residual or paralyzed limbs cannot operate conventional myoelectric hands, which are designed to detect muscle activity in residual limbs [12,16]. Electrodes placed in residual parts of the limbs may aggravate pain in individuals with upper-limb deficiencies and sensory disturbances.

To overcome these problems, we developed a novel electric hand prosthesis that does not require muscle activation in residual limbs and is economical and lightweight. This project involved a multidisciplinary medical team that included physiatrists, orthopedic surgeons, physical therapists, occupational therapists, a prosthetist, and engineers, who incorporated feedback from users. Therefore, this study aimed to compare this novel prosthetic hand with a conventional myoelectric hand in healthy participants and evaluate the feasibility and safety of our novel hand.

Our first objective was to develop a sensor not based on myoelectricity for an upper-limb prosthesis, focusing on shape deformation sensing. The bioinstrument utilizes a bridge circuit and multiple amplifier circuits with strain gauges [17]. Concerns regarding increased weight, cost, and heat generation have led to the adoption of a flexible sensor, thus reducing electronic parts and addressing overheating issues [18]. However, these sensors were insufficient in terms of accuracy and reproducibility and did not adequately reduce the weight of the hand. Consequently, to allow for more precise and intuitive hand movements while minimizing weight, the use of a wireless sensor was explored.

Developing wireless sensing technology was initially a formidable technical challenge, but it was eventually realized after several iterations by our medical engineering team. We opted to use a wireless sensor button in our novel hand, offering an alternative for individuals with upper-limb deficiencies who suffer from paralysis or sensory disturbance, as this system eliminates the need to place the sensor on the hand or limbs.

Furthermore, we investigated an optimal prosthetic hand design, acknowledging that prosthetic hands have a high abandonment rate [2,13]. As a result, we developed new electric prosthetic hands based on users’ perspectives such that they could use the prostheses comfortably. Finally, this study focused on maintaining socket fittings. This is because myoelectric sensors must be in proper contact with the skin in myoelectric hand prostheses. Some myoelectric hand users have skin problems with myoelectric sensors. As such, a method is proposed to allow the development of a wearable, novel wireless prosthetic hand without using electric sensors to improve socket fittings. Our novel hand design incorporates a wireless sensor button to flex and extend fingers without the sensor contacting the residual limbs.

## 2. Materials and Methods

### 2.1. Novel Hand Based on Users’ Perspectives

#### 2.1.1. Users’ Perspectives

Before constructing a prototype of the electric hand, we investigated users’ perspectives on prosthetic hands through in-person interviews with five male upper-limb amputees and by administering relevant questionnaires (Table 1). Based on the obtained results, we discussed the optimal style for the prosthetic hand.

All participants acknowledged the necessity of prosthetic hands, underscoring their significance for ADL and work. None expressed hesitation in using a prosthesis, indicating a positive attitude towards these assistive devices. The participants in this study are described as follows.

User 1, a man in his 40s with a humeral-level amputation on the right side, had been prescribed body-powered cosmetic and myoelectric prostheses. His primary concerns with the myoelectric prosthesis were malfunctions, increased weight, and cost, which were particularly troubling due to his history involving an industrial accident. For his daily activities, light work, office tasks, and appearance were the most critical factors, followed by the function and weight of the prosthesis. His hand function priorities were wrist-angle adjustment, grip, and pinch.

User 2, a man in his 20s with a forearm-level amputation on his left side, preferred a hooked prosthesis over a myoelectric hand because of its lighter weight, which was beneficial for his work that involved carrying heavy loads. He also utilized cosmetic and body-powered prostheses. His priorities decreased in order of function, appearance, and weight, indicating a practical approach to his prosthetic needs. Similar to User 1, his hand function priorities were wrist-angle adjustment, grip, and pinch.

User 3, a man in his 30s with a forearm-level amputation on his right side, identified cost as the sole issue with the myoelectric hand. His prosthesis use was for daily activities and tasks requiring fine motor skills, such as calligraphy. He prioritized the function of the prosthesis, followed by weight and then appearance, with his hand function priorities being wrist-angle adjustment, grip, and pinch.

User 4, also a man in his 30s but with a forearm-level amputation on the left side, faced similar concerns about the cost of the myoelectric hand. He used a prosthesis for daily life and office work, valuing function and appearance equally, and then weight. His hand function priorities were grip, wrist-angle adjustment, and pinch.

User 5, a man who was similar in age and level of amputation to User 1 but on his left side, encountered issues with the myoelectric hand related to malfunction, increased weight, and cost. His prosthetic use was primarily for daily life and light work, preferring the body-powered type. He valued function and appearance equally, followed by weight, and his priorities of hand function were wrist-angle adjustment, grip, and pinch.

The diverse backgrounds and experiences of these users highlight the multifaceted needs of prosthetic hand users, which span functional requirements and personal preferences regarding appearance, weight, and specific hand functions such as grip and wrist movement.

#### 2.1.2. Prosthetic Hand Design

**Figure 1:**
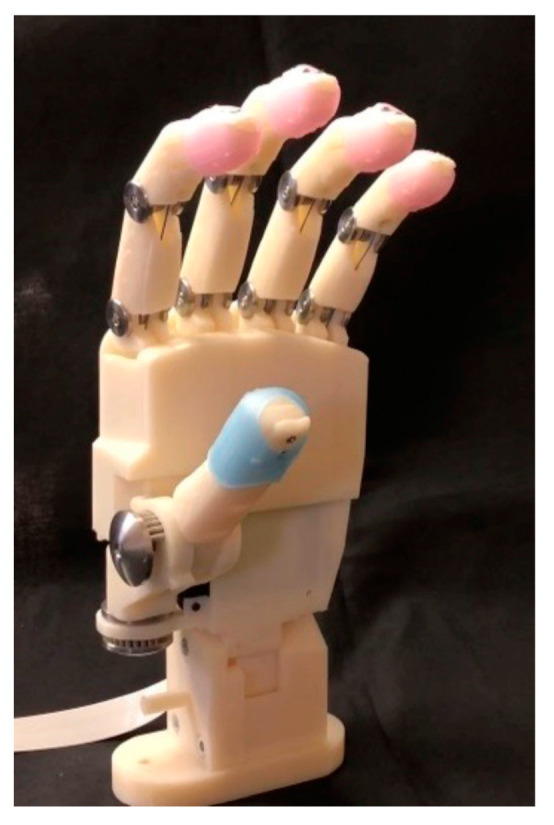
Novel wireless prosthetic hand. The proposed hand is constructed predominantly from nylon resin and shaped using a three-dimensional (3D) printer, with stainless steel employed for the joints to enhance durability. It contains two linear actuators within its structure that control the flexion and extension of each finger, from the index to the little finger, via fishing lines. Externally, stainless-steel mechanisms facilitate the radial-palmar abduction and adduction movements of the thumb.

**Figure 2:**
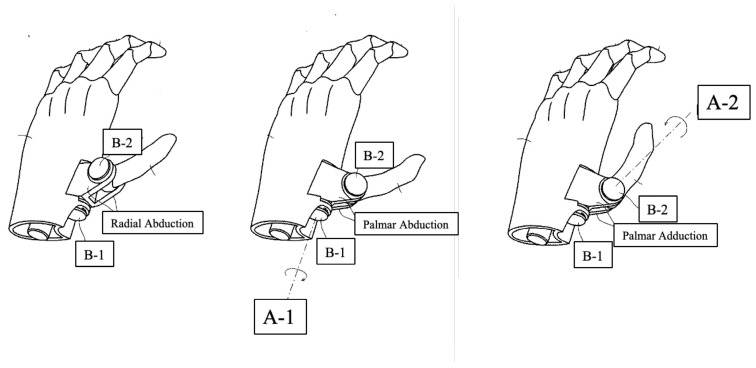
Schematic of our novel wireless prosthetic hand.

An optimal prosthetic hand design was developed based on user feedback. The conventional Ottobock hand (OH) is a three-finger pinch-type prosthesis; however, users expressed a preference for gripping over pinching. Focus was also directed towards wrist function within the prosthetic hand design. During ADL tasks, such as pressing down on a paper or holding objects, users need to adjust their wrist flexion angle, which decreases compensatory movements from the other parts of the body. Consequently, it was determined that the ideal prosthetic hand style should facilitate gripping, grasping, and pinching, as well as adjusting wrist flexion angles. In collaboration with Iwata Machinery Works Ltd. (Gifu, Japan), we developed a novel wireless prosthetic hand that supports gripping, grasping, and pinching while maintaining lightness (Figure 1 and Figure 2). The prosthetic hands were primarily composed of a nylon resin produced by a three-dimensional (3D) printer, effectively reducing the overall weight. For increased durability, stainless steel was integrated into the joint construction. The four digits, from the index to the little finger, are actuated simultaneously through traction, which is achieved using two linear actuators connected to fishing wires. One linear actuator is connected to the index and middle fingers, and the other to the ring and little fingers. Instead of utilizing a pulley system, each finger’s flexion wire runs along the finger midsection from the tip to the base. Linear movement of the actuator exerts force on the fingertip, causing the finger segment to flex. Relaxation of the flexion wire as the actuator retracts extends the finger. The thumb’s movement is manually controlled by two single-push switches, which are connected to the carpometacarpal and metacarpophalangeal joints, respectively, allowing six distinct positions for refined manipulation.

The four digits, from the index to the little finger, are actuated simultaneously by two linear actuators connected to fishing wires, facilitating traction-based movement. Each actuator controls two fingers, enabling both flexion and extension actions without the use of pulleys. As the actuators move, they cause the fingers to flex and extend. The thumb is operated manually via two single-push switches, influencing the carpometacarpal and metacarpophalangeal joints. These joints rotate around two axes: A-1 for radial abduction and A-2 for palmar abduction. Pressing switch B-1 initiates radial abduction, while pressing B-2 leads to palmar abduction, with the ability to achieve additional thumb positions such as radial adduction (as shown in Figure 2). These schematics are informed by the Japan Platform for Patent Information: JP6953007B [19].

### 2.2. Wireless Button Sensor

A wireless button sensor was devised using a radiofrequency (RF)-based wireless system (nRF24L01, Nordic Semiconductor Inc., Trondheim, Norway, 2010 [20]). Two buttons were externally mounted on the plastic container, while the wireless system itself was housed internally. These buttons can be positioned as per user preference; in this study, they were placed on the contralateral trunk side in line with the experimental conditions of the conventional OH.

Operation of the wireless button requires users to continuously press one button for gripping and the other for releasing the prosthetic hand. Pressing a button activates the linear actuator to operate at its maximum velocity. However, when the fingers reach their maximum range of motion, they are compelled to remain still to safeguard the actuator.

**Figure 3:**
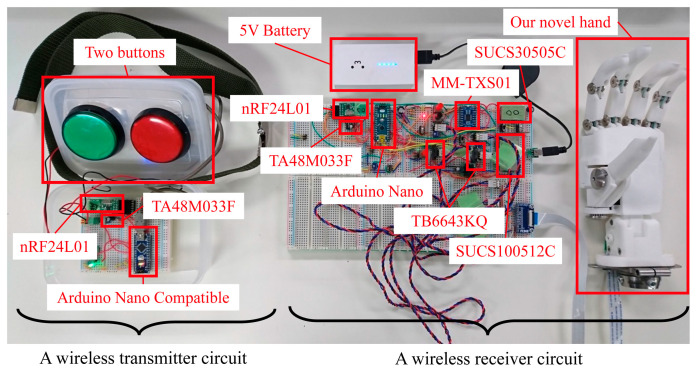
Photograph showing all the systems designed and tested in this study.

The communication module is an RF-based, ultracompact wireless module (nRF24L01), utilizing Nordic’s NRF24L01 2.4 GHz band transceiver chip. It is compact, cost-effective, power-efficient, and microcomputer-compatible. Operating on the 2.4 GHz ISM frequency band, it features power and receiving amplifiers and switches between the transmission and reception modes using appropriate circuitry in the chip. Combined with a microcontroller, this chip can directly control the radio, and the output can be programmed. Other module specifications were as follows: 126 RF channels; signal strength: 0/−6/−12/−18 dBm; current consumption: 7 mA@−18 dBm; 11 mA@0 dBm; operating power: 1.9 to 3.6 VDC; maximum data rate: 1 Mbps/2 Mbps (on-air data rate). Figure 3 shows the system comprising an Arduino Nano, the nRF24L01 module, and our novel hand. On the left side is the wireless transmitter circuit with a wireless module nRF24L01, which is compatible with the Arduino Nano, a low-loss 3-terminal regulator TA48M033F with an output voltage of 3.3 V and an output current of 500 mA (maximum), and two buttons. The right side is a wireless receiver circuit with the wireless module nRF24L01, Arduino Nano, motor driver IC TB6648KQ, DC/DC converter SUCS30505C (rated output wattage 3 W, rated input voltage 5 V, rated output voltage 5 V), DC/DC converter SUCS100512C (rated output wattage 10 W, rated input voltage 5 V, rated output voltage 12 V), low-loss 3-terminal regulator TA48M033F, dual-supply 8-bit bidirectional IC (MM-TXS01 with TXS0108E, TXS0108E, Texas Instruments Inc., Dallas, TX, USA) capable of bidirectional logic-level conversion between 1.2 and 5.5 V, and our novel hand with two linear actuators (PQ12-100-12-P; power supply voltage: 12 VDC; gear ratio: 100:1; maximum speed (no load): 10 mm/s; peak power: 40 N at 6 mm/s; 3.3 V potentiometer, Actuonix Motion Devices Inc., Saanichton, BC, Canada [21]).

**Figure 4:**
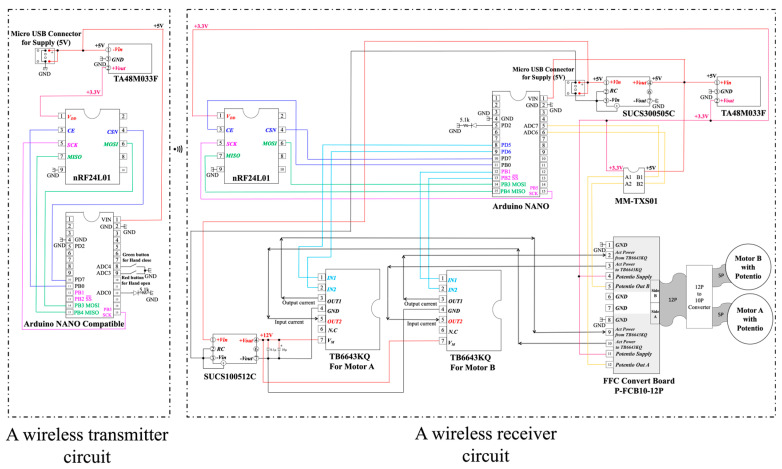
Schematic of the wireless receiver circuit. The wireless transmitter circuit on the left side and a schematic of the wireless receiver circuit on the right side. In the wireless transmitter circuit, the 5 V battery directly supplies 5 V to the Arduino NANO compatible, and the dropout 3.3 V is supplied to the nRF24L01 via a low-loss 3-terminal regulator TA48M033F. While the green button is pressed, ADC4 goes low, and the hand can be operated to open. On the other hand, while the red button is pressed, ADC3 goes low, and the hand can be operated to close. If both are pressed or neither is pressed, the hand stops. In the wireless receiver circuit, the voltage from the 5 V battery is converted to a safe 5 V isolated from the battery via a DC/DC converter SUCS30505C, and then the dropped 3.3 V is supplied to the nRF24L01 via a three-terminal regulator TA48M033F. The voltage of the 5 V battery is boosted to the required 12 V via the DC/DC converter SUCS100512C, and then the 12 V is used to drive two linear actuators PQ12-100-12-P through two motor drivers IC TB6648KQ.

The gripping force of a prosthetic hand can be adjusted via a PQ12-100-12-P linear actuator by altering its speed or current, providing a linear force–speed/current relationship. This actuator provides a force ranging between 0 and 40 N. The maximum grip force of the hand, which is influenced by the actuator and the finger structure of the hand, is limited to less than 40 N. Figure 4 shows a schematic of the wireless transmitter circuit on the left side and a schematic of the wireless receiver circuit on the right side. In the wireless transmitter circuit, the 5 V battery directly supplies 5 V to the Arduino NANO Compatible, and the dropout 3.3 V is supplied to the nRF24L01 via a low-loss 3-terminal regulator TA48M033F. However, when the green button is pressed, ADC4 becomes low, enabling the hand to open. However, when the red button is pressed, ADC3 becomes low, enabling the hand to close. If both are pressed or neither is pressed, the hand stops.

Left: a wireless transmitter circuit with the wireless module nRF24L01, Arduino Nano-compatible module, low-loss 3-terminal regulator TA48M033F with an output voltage of 3.3 V and output current of 500 mA (maximum), and two buttons. Right: a wireless receiver circuit with the wireless module nRF24L01, Arduino Nano, motor driver IC TB6648KQ, and DC/DC converter SUCS30505C (rated output wattage 3 W, rated input voltage 5 V, rated output voltage 5 V), DC/DC converter SUCS100512C (rated output wattage 10 W, rated input voltage 5 V, rated output voltage 12 V), low-loss 3-terminal regulator TA48M033F, dual-supply 8-bit bidirectional IC (MM-TXS01 with TXS0108E, Texas Instruments Inc.) capable of bidirectional logic level conversion between 1.2 and 5.5 V, and our novel hand with two linear actuators (PQ12-100-12-P; power supply voltage: 12 VDC; gear ratio: 100:1; maximum speed (no load): 10 mm/s; peak power: 40 N at 6 mm/s; 3.3 V potentiometer, Actuonix Motion Devices Inc., Saanichton, BC, Canada) inside.

In the wireless receiver circuit, the voltage from the 5 V battery is converted to a safe 5 V isolated from the battery via the DC/DC converter SUCS30505C, and then the dropped 3.3 V is supplied to the nRF24L01 via a three-terminal regulator TA48M033F. The voltage of the 5 V battery is boosted to the required 12 V via the DC/DC converter SUCS100512C, and then the 12 V is used to drive two linear actuators PQ12-100-12-P through two motor drivers IC TB6648KQ.

All members of the research team confirmed the effect of the time delay associated with the response after pressing the wireless button in a preliminary experiment and determined that it would not interfere with the execution of the experiment.

### 2.3. Participants and Method

#### 2.3.1. Participants

Ten non-disabled therapists were enrolled in this study: five men and five women, including six physical therapists and four occupational therapists, with an average age of 29.7 ± 4.9 years. The inclusion criteria were as follows: (1) absence of any neurological or motor impairments; (2) normal vision; and (3) lack of experience with prosthetic simulation training. The Human Ethics Review Committee of the Ibaraki Prefectural University of Health Sciences approved the study protocol (approval No. 817 from 20 May 2018, e191 from 29 November 2018, and e305 from 21 February 2021). This study was conducted in accordance with the Declaration of Helsinki principles. All participants provided written informed consent.

#### 2.3.2. Materials

**Figure 5:**
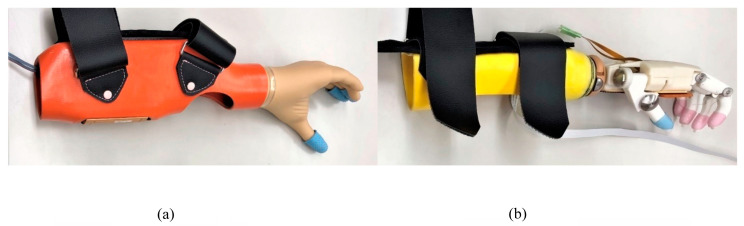
Prosthetic simulator hands. Two prosthetic simulators were developed to compare both hands. The body of the simulators was made of fiber-reinforced plastic (FRP), where acrylic resin was infiltrated into nylon, cotton, and carbon fiber layers. (**a**) Ottobock hand (8E38 = 6 DMC plus, Ottobock, Germany) with the simulator. The simulator was made of FRP and leather belts such that it could fit the user’s forearm while using the Ottobock hand. It also had a special battery for use with only the Ottobock myoelectric hand. Non-slip finger sacs were worn on the fingers. (**b**) Our novel hand and wrist component for cosmetic hands (10V39 and 10A30, Ottobock, Germany) with the simulator hand. The simulator was also made of FRP and leather belts such that it could fit the user’s forearm while using the Ottobock hand. Non-slip finger sacs were worn on the fingers.

**Figure 6:**
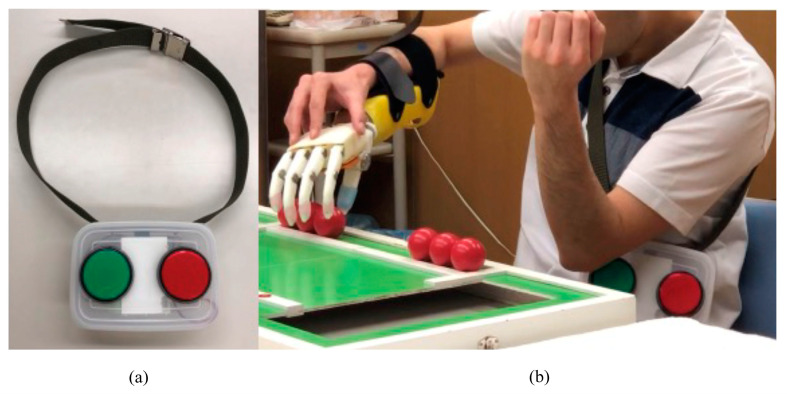
Wireless sensor with control buttons. (**a**) Photograph showing the two buttons outside the plastic container. (**b**) A participant, who is in the middle of the STEF test using the novel hand, places the control button box on the left trunk while the novel hand is worn on the right forearm. The green button is a trigger for four-finger flexion; the red is for extension.

For comparison purposes, a prosthetic simulator was designed for both the novel wireless electric prosthetic hand and a conventional myoelectric hand (Myobock system with System Electric Hand DMC Plus, Ottobock, Duderstadt, Germany) (Figure 5). The simulators’ bodies were constructed from fiber-reinforced plastic (FRP), which was created by embedding resin into laminated layers of nylon and cotton fibers and then reinforced with carbon fiber around the wrist component. Each simulator was affixed to the right forearms of the participants, and triggers for the hands were positioned on the left side of both arms. The OH was equipped with myoelectric sensors for finger flexion on the left elbow flexor and for finger extension on the left elbow extensor. Conversely, the NW featured a wireless button sensor placed on the left trunk side (Figure 6). Additionally, finger stalls were affixed to the digits of both hands.

**Figure 7:**
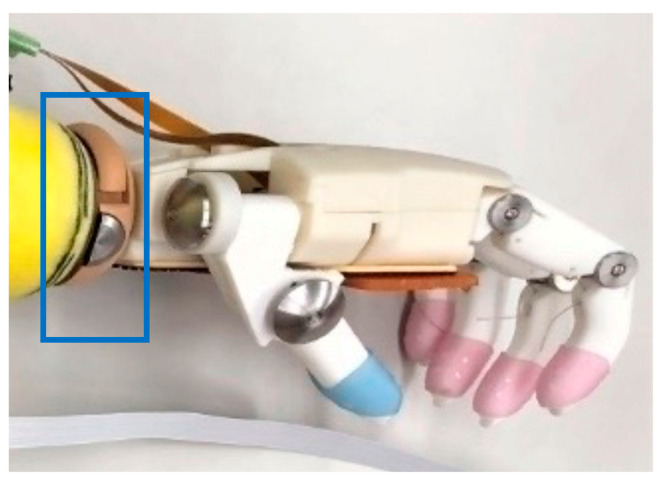
Ottobock wrist part in our prosthetic simulator hand. Ottobock wrist parts (10V39 and 10A30) for a cosmetic hand (in a blue square) are used for our novel hand.

This hand simulator for our novel hand system was created using the Ottobock wrist parts (10V39 and 10A30); these are common prosthetic wrist parts that can flex the wrist and rotate the forearm. Our novel hand was designed to be compatible with existing cosmetic hands (Figure 7). Our hand weighed approximately 375 g (with the simulator), which is approximately 100 g lighter than OH (the weight, including that of the simulator, is 470 g).

#### 2.3.3. Clinical Evaluation

##### Upper-Limb Function

Each hand was clinically evaluated using the Simple Test for Evaluating Hand Function (STEF) [22,23] and the Action Research Arm Test (ARAT) [24,25]. The STEF is used to evaluate a patient’s ability to pinch, grasp, and transfer objects. During this test, the subject was instructed to pick up items (one by one) from a storage space and move them into the target space as fast as possible. The subject performed the object-moving tests using 10 types of objects with different shapes and sizes, including (1) five large balls (7 cm in diameter), (2) six middle-sized balls (4 cm in diameter), (3) five large cuboids (5 cm long, 10 cm wide, and 10 cm tall), (4) six middle-sized cubes (3.5 cm per side), (5) six wooden circular disks (3 cm in diameter and 1 cm thick), (6) six small cubes (1.5 cm per side), (7) six pieces of cloth (9 cm long and 7 cm wide), (8) seven metallic circular disks (2 cm in diameter and 0.2 cm thick), (9) six small balls (0.5 cm in diameter), and (10) eight pins (0.3 cm in diameter and 4 cm long). The total score was 100 points, and the scores were calculated based on the completion time for each subtest, with higher scores indicating superior hand function. This method, developed in Japan, is one of the most common hand function evaluation methods. The ARAT, the most extensively used standardized measurement test for upper limbs, is efficient and can assess both the arm and hand during the execution of functional tasks. It assesses four groups of motion types (grasp, grip, pinch, and gross movements) that incorporate bimanual ADLs: (a) grasping (six subscales: 2.5, 5, 7.5, and 10 cm blocks, cricket ball, and sharpening stone), (b) gripping (four subscales: pouring water from one glass to another, displacement of an alloy tube from one side of the table to the other by 2.25 cm, displacement of an alloy tube from one side of the table to the other by 1 cm, and placement of a washer over a bolt), (c) pinching (six subscales: ball-bearing held between the ring finger and thumb, marble held between the index finger and thumb, ball-bearing held between the middle finger and thumb, ball-bearing held between the index finger and thumb, marble held between the ring finger and thumb, and marble held between the middle finger and thumb), and (d) gross movement (three subscales: hand behind the head, hand at the top of the head, and hand on the mouth). The examiner demonstrates each task to each participant, and they perform each task using an upper limb with the simulator hand. The examiner scores the performance based on a four-point scale (0: no movement; 1: partial movement; 2: completion with compensatory movement; 3: normal movement). The most challenging task was assessed first, followed by progressively easier tasks. The total score ranged from 0 to 57. All participants were randomly allocated to the OH or NW groups. The training time was set to be below 10 min for each hand. Both test results were scored by a test-experienced occupational therapist, a physical therapist, and a physiatrist.

##### Fatigue While Using the Simulator Hand

The degree of fatigue was also evaluated using the modified Borg scale [26,27], which is a numerical scale that measures perceived exertion or breathlessness. It typically ranges from 0 to 10, with 0 indicating no exertion at all and 10 indicating maximum exertion or breathlessness. This scale is helpful in assessing the intensity of physical activity and an individual’s subjective exertion or breathlessness. The participants were asked to rate their degree of fatigue before each hand experiment.

#### 2.3.4. Statistical Analysis

As all data including the ARAT score, STEF score, and modified Borg scale were ordinary scales, all statistical analyses were conducted using the Wilcoxon signed-rank test, and *p*-values less than 0.05 were considered statistically significant. Analyses were performed using the software JMP (version 14.3.0, SAS Institute Inc., Cary, NC, USA).

## 3. Results

### 3.1. ARAT

No significant differences were identified in the ARAT total scores between the OH and NW groups. The participants could not pinch the 6 mm ball using the NW; however, some of them could use the OH. The median scores for OH and NW were 6 and 3, respectively (*p* = 0.0039). Table 2 summarizes the ARAT results, and the results for all subjects are presented in Supplementary Table S1.

### 3.2. STEF

No significant differences were observed in the total STEF scores between the OH and NW groups. Additionally, no significant differences were noted in the subscore of the six wooden circular disks between the OH and NW groups (the median scores of OH and NW were zero and 0.5, respectively, at *p* = 0.063).

The eighth to tenth subscores on both hands resulted in no scores. The STEF results are listed in Table 3, and the results for all the subjects are presented in Supplementary Table S2.

### 3.3. Modified Borg Scale

NW showed a lower Borg scale change than OH (the median scores of both OH and NW were 1; *p* = 0.045), and the results for all the subjects are presented in Table 4 and Supplementary Table S3.

No adverse events were observed throughout the experiment when either hand was used.

## 4. Discussion

We have developed a novel wireless prosthetic hand that is tailored to the users’ needs and preferences, offering intuitive control that allows users to grip and pinch objects by pressing a wireless button—a significant advantage over traditional myoelectric hands. By using wireless sensor technology, our hand has the ability to reduce prosthetic hand weight and involves intuitive button-pressing, and advantageous sensor placement can be selected. Our hand provides a viable alternative for individuals with upper-limb deficiencies, particularly those with short, paralyzed, or sensory-impaired residual limbs who may find conventional myoelectric hands unsuitable. Although our hand did not demonstrate statistically significant differences in overall upper-limb function when compared to the globally used OH, we identified the need for enhancement in its pinching function through ARAT and STEF assessments. Reflecting the feedback from the users, this aspect will be a focus for further development.

Our prosthetic hand has several important characteristics. First, muscle activity is not required for its use. This prosthetic hand can also be used by people with paralysis in residual limbs and people with short residual limbs, which is a potential option for amputees with severe phantom limb pain.

Our design also prioritizes ease of rehabilitation and aims to shorten the adaptation period for new users. Myoelectric hands require a certain training period [28,29,30]. Most upper-limb amputees often have a strong desire to resume their social activities as soon as possible following their injuries as they can still walk and perform daily activities using the uninvolved side. Therefore, we wanted to shorten their rehabilitation periods using our novel prosthetic hand.

Second, our prosthetic hand has a wireless sensor that can be placed wherever the user desires. High-level amputees often lack residual muscle. This can constitute an obstacle in terms of using myoelectric hands [17]. While conventional myoelectric hands are associated with the risk of malfunction, which occurs owing to improper fittings of the electrodes to the residual limbs [17,30], our users did not have to worry about malfunctions when using our wireless button system. According to User 1, the greatest mechanical and psychological barrier to using a myoelectric hand was the possibility of malfunction. In this preliminary study of healthy individuals, participants were able to intuitively control the hand by pressing the wireless buttons without training or fear of malfunction. This wireless sensor will mitigate the risks associated with myoelectric hand malfunction due to improper electrode fitting and requires no training, thus addressing the mechanical and psychological barriers expressed by users. Additionally, the wireless button system enables users to set the button apart from the residual limbs (e.g., closer to the feet rather than the surrounding upper limbs), depending on the user’s preferences. In this pilot study, we set the wireless button on the trunk of the contralateral side. However, having more options for the location of the wireless wearable sensor allows users to focus on bimanual actions, which is particularly beneficial for beginners who find it challenging to concentrate on both the prosthetic hand operation and other procedures simultaneously. Our ultimate goal is to make the wireless sensor wearable in the future such that pressing the button does not avoid the use of the contralateral side. Additionally, the pressing action does not interfere with daily activities such as talking or eating. Furthermore, users can easily select where to wear the button. In this study, the wireless button sensor was only utilized for the evaluation of our novel hand compared to the OH.

Third, the finger design of our prosthetic hand was developed with the users’ desires in mind. They desired to have the ability to grip, grasp, and pinch. Functional five-finger hands exist for clinical use, such as the Michelangelo hand (Ottobock, Germany) [31,32], the i-Limb Quantum (Ossur, Iceland), and the bebionic hand EQD (Ottobock, Germany), but they are all expensive. Without public support, they cannot be provided to most people with upper-limb deficiencies. Moreover, considering the rejection and abandonment rate of prosthetic hands [2,7,8,11], it is crucial to address the unmet needs of upper-limb amputees. As stated in previous research, the co-creation of prosthetics by academics, clinicians, users, and industry personnel is an excellent approach to the development of new upper-limb prostheses [33,34]. From this perspective, our multidisciplinary medical engineering research team searched for the optimal prosthetic hand design.

Our first objective was to develop a sensor that was not based on myoelectricity to broaden the scope for users who could leverage its functionality and features. Therefore, we developed an electric upper-limb prosthesis that functions based on the sensing of shape deformation in the residual limb. First, we used a skin sensor within a specified range of output voltages in conjunction with multiple strain gauges placed on the soft and stretchable skin surface [17]. Second, we applied a flexible sensor to this system instead of strain gauges. Accordingly, the total number of electronic parts could be reduced substantially. Furthermore, the flexible sensor can prevent overheating owing to an overcurrent because the sensor has a tremendously large resistance [18]. However, to accommodate user preferences, it is necessary to substantially lighten the developed hand. Upon careful consideration, we realized that separating the sensor from the main body of the hand could be a viable solution. This insight eventually guided us to the adoption of a wireless sensor, a decision that significantly contributed to the overall weight reduction of the developed hand.

We also chose 3D printing methods to mitigate the hand weight. This technology is a promising new method for developing prostheses [35], and our team strived to develop optimal hand prostheses using this technology. To achieve this, our team of clinicians, including physiatrists, physical and occupational therapists, and a prosthetist, collaborated to devise optimal finger and hand designs. Based on these, our engineers developed and modified sensors, and a manufacturer constructed the hand using 3D printing. Nylon resin was used for printing the fingers, and stainless steel was incorporated into the joints to increase strength. To enable simultaneous gripping and pinching, the thumb’s motion was set independently from the other four fingers. In addition, as our hand received scores lower than those of the OH in terms of the participant’s ability to pinch a 6 mm ball in the STEF, we decided to expend additional efforts to improve this functional aspect.

Based on our users placing high priority on the ability to move their prosthetic wrists manually, we considered developing new wrist joints. However, we finally chose the Ottobock wrist components for conventional cosmetic hands or hooked-type hands (10V39 and 10A30) because of their dorsiflexion, palmar flexion, and rotation capabilities. According to a previous report, prosthetic manual wrists are functionally related to prosthetic hands [36], and users with existing wrist joints can easily fit our novel hand over them. Current prosthetic wrists are incompatible with body-powered, cosmetic, and myoelectric hands; therefore, our developed hand’s compatibility can enhance the users’ quality of life. This is also because we prioritized hand evaluation over wrist function, which allowed us to utilize an existing wrist joint.

Finally, based on the end users’ perspectives, our developed hand was approximately 100 g lighter than the OH, which may be one of the reasons for the differences in the Borg score changes documented throughout the experiment. Increased weight, which is related to fatigue or neck and shoulder pain, is one of the factors related to the high abandonment rate of prosthetic use [8,13]. Our novel hand may potentially reduce the users’ workloads.

However, there are significant barriers to developing a myoelectric prosthetic hand. The OH is robust and powerful, but heavier than our developed hand. Our hand, in contrast, needs more power and endurance. Myoelectric hands typically require considerable time for training to contract the muscles in the user’s residual limbs; however, our hand design is more intuitive and may experience fewer malfunctions. One of the most vital features of our hand is that it can be used and combined with existing cosmetic hands, as it uses the same wrist part as these hands. Users of cosmetic hands can readily test our new electric hand, as it is designed to fit seamlessly into the sockets of their existing prostheses. Using the same wrist part as that used in a cosmetic hand provides people with upper-limb deficiency easy accessibility to electric hands. Our multidisciplinary prosthetic hand team aims to mitigate financial, mechanical, and psychological barriers for people with upper-limb deficiencies to help them obtain better prosthetic hands.

This study has certain limitations. First, this study was conducted using a small group of participants who were experts in rehabilitation. The selection of participants was a limitation; therefore, we intend to conduct experiments by engaging people with upper-limb deficiencies based on this pilot study. In future experiments for individuals with upper-limb deficiencies, we should confirm the potential of our prosthetic hand to prevent malfunctions. Second, the ARAT and STEF have not been developed for evaluating people with upper-limb deficiencies. There are other dedicated tests for evaluating people with limb deficiencies using prostheses. The assessment of capacity for myoelectric control should only be performed by specialists who have completed training [37]. The Southampton Hand Assessment Procedure [38] is designed specifically for prosthetic use, but it needs special tools for evaluation. Both the ARAT and STEF are common evaluation tools for upper-limb function that many rehabilitation specialists can use easily and repeatedly. However, we consider that using optimized tests is more appropriate for evaluating our novel hand for research studies involving prosthetic users. Finally, our hand must be improved in terms of its pinch functionality, robustness, and selection of optimal sites for wireless buttons. Additional research is required to improve these possibilities.

Our novel prosthetic hand focused on affordability and accessibility compared with the traits of highly functional and expensive prosthetic hands without sacrificing functionality; rather, priority was placed on all the indispensable functions of prostheses for people with upper-limb deficiencies.

In this study, a novel wireless prosthetic hand was proposed, and its performance was evaluated. Based on this preliminary research study, the development of this prosthetic hand as a practical product will be continued.

## 5. Conclusions

In this study, we developed a novel wireless prosthetic hand based on users’ perspectives. Our hand can be driven intuitively, thus allowing gripping and pinching by pushing a wireless button. Compared to the existing OH, our developed prosthetic hand shows potential as an innovative solution for upper-limb amputees and is especially suitable for individuals with short, paralyzed, or sensory-impaired residual limbs, as well as for those who cannot use conventional myoelectric hands.

## Figures and Tables

**Table 1:** Users’ perspectives of prosthetic hands.

User	1	2	3	4	5
Age group	40s	20s	30s	30s	40s
Level	Humerus	Forearm	Forearm	Forearm	Humerus
Right/left	Right	Left	Right	Left	Left
Necessity	Yes	Yes	Yes	Yes	Yes
Hesitation in using a prosthesis	No	No	No	No	No
Type of prosthesis	Body-powered,	Body-powered,	Body-powered,	Body-powered,	Body-powered,
	cosmetic, myoelectric	cosmetic, hooked	cosmetic, myoelectric	cosmetic, myoelectric	cosmetic, hooked
Myoelectric hand problem	Malfunction,	Increased weight, cost	Cost	Cost	Malfunction,
	increased weight, cost				increased weight, cost
Objectives	Daily life,	Daily life,	Daily life,	Daily life,	Daily life,
	light work, office work	carrying heavy loads	light work, Calligraphy	light work, office work	light work
Priority	1. Appearance	1. Function	1. Function	1. Function	1. Function
	2. Function	2. Appearance	2. Weight	1. Appearance	1. Appearance
	3. Cost	3. Cost	3. Appearance	3.Weight	3. Cost
Priority in hand function	1. Wrist angle adjustment	1. Wrist angle adjustment	1. Wrist angle adjustment	1. Grip	1. Wrist angle adjustment
	2. Grip	2. Grip	2.Pinch	2. Wrist angle adjustment	2. Grip
	3. Pinch	3. Pinch	3. Grip	3. Pinch	3. Pinch

**Table 2:** Action Research Arm Test (ARAT) score results.

	Grasp		Grip		Pinch		Gross motor		Total	
	OH	NW	OH	NW	OH	NW	OH	NW	OH	NW
Median	15	15	12	12	6	3	9	9	71	66
IQR	14.5–15	14–15	11–12	12	4–6	3	9	9	67–75	63–66
	*p* = 1.00		*p* = 0.75		※ *p* = 0.0039		*p* = 1.00		*p* = 0.29	

OH: Ottobock hand; NW: our novel hand; IQR: interquartile range. ※ indicates that the result is statistically significant with *p* < 0.05.

**Table 3:** Simple Test for Evaluating Hand Function (STEF) score results.

	**1**		**2**		**3**		**4**		**5**	
	Five large balls		Six middle-sized balls		Five large		Six middle-sized cubes		Six wooden	
					cuboids				circular disks	
	OH	NW	OH	NW	OH	NW	OH	NW	OH	NW
Median	1	1	1	2	0	0.5	0	0	0	0.5
IQR	0.75 –3.5	0.75–1.0	0–2	0–2.25	0–1	0–1	0–0.25	0–1	0	0–2
	*p* = 0.13		*p* = 0.57		*p* = 0.53		*p* = 0.81		*p* = 0.063	
	**6**		**7**		**8, 9, 10**				**Total**	
	Six small cubes		Six pieces of clothes		Seven metallic circular disks,					
					Six small balls, eight pins					
	OH	NW	OH	NW	OH		NW		OH	NW
Median	0	0	0	0.3	0		0		3	5
IQR	0	0–1.25	0.1	0–1	0		0		1.5–6.25	1–8.5
	*p* = 0.13		*p* = 0.50		*p* = 1.00		*p* = 1.00		*p* = 0.26	

OH: Ottobock hand; NW: our novel hand; IQR: interquartile range.

**Table 4:** Summary of comparisons between two hands.

	ARAT		STEF		Change of Borg Scale	
	OH	NW	OH	NW	OH	NW
Median	71	66	3	5	1	1
IQR	67–75	63–66	1.5–6.25	1–8.5	1–1.375	0–2.125
	*p* = 0.29		*p* = 0.26		※ *p* = 0.045	

OH: Ottobock hand; NW: our novel hand; IQR: interquartile range. ※ indicates that the result is statistically significant with *p* < 0.05.

## Data Availability

The datasets generated for this study can be found in Supplementary Tables S1 and S2.

## Supplementary Materials

The following supporting information can be downloaded at: https://www.mdpi.com/article/10.3390/s24092765/s1, Table S1: Action research arm test (ARAT) score, title; Table S2: Simple test for evaluating hand function score, Table S3: Borg Scale Change. The movie shows the functions of pinching, grasping, and gripping. In addition, video clips from a participant performing the 5th STEF (moving six wooden disks) are shown.
