# Supplementary material for: Developing a Novel Prosthetic Hand with Wireless Wearable Sensor Technology Based on User Perspectives: A Pilot Study
Source: Sensors (Basel). 2024 Apr 26;24(9):2765. doi: 10.3390/s24092765 (PMC11086240; doi:10.3390/s24092765)
Supplement: Supplementary file 1 [file sensors-24-02765-s001.zip › sensors-2946259-supplementary.pdf]

sensors MDPI

Supplementary Table S1. Action research arm test (ARAT) score (Total 57)

Participant	Grasp		Grip		Pinch		Gross motor		Total	
	OH	NW	OH	NW	OH	NW	OH	NW	OH	NW
1	15	12	12	12	4	3	9	9	71	60
2	13	14	12	12	6	3	9	9	71	64
3	15	15	5	12	6	3	9	9	61	66
4	15	14	12	7	4	3	9	9	71	54
5	15	14	12	12	3	3	9	9	69	64
6	15	15	12	12	6	3	9	9	75	66
7	15	15	12	12	5	3	9	9	73	66
8	11	15	8	12	6	3	9	9	59	66
9	15	15	12	12	6	3	9	9	75	66
10	15	15	12	12	6	3	9	9	75	66
**Median**	15	15	12	12	6	3	9	9	71	66
**IQR**	14.5-15	14-15	11/12/2024	12	4/6/2024	3	9	9	67-75	63-66
	P = 1.00		p = 0.75		※p = 0.0039		p = 1.00		p = 0.29	

OH: Ottobock hand, NW: Our novel hand, IQR: Interquartile range.

Supplementary Table S2. Simple test for evaluating hand function score (Total 100).

Participant	1		2		3		4		5	
	Five large balls		Six middle-sized balls		Five large cuboids		Six middle-sized cubes		Six wooden circular disks	
	OH	NW	OH	NW	OH	NW	OH	NW	OH	NW
1	0	0	0	0	0	0	0	0	0	0
2	3	1	2	0	1	0	1	0	0	0
3	1	1	2	3	0	1	0	1	0	1
4	1	1	1	0	0	0	0	0	0	0
5	3	1	0	2	0	0	0	0	0	1
6	1	1	2	4	0	2	0	1	0	2
7	0	1	0	2	0	1	0	0	0	2
8	5	3	4	1	1	0	2	0	0	0
9	5	0	0	2	1	1	0	3	0	3
10	1	1	1	2	0	1	0	0	0	0
**Median**	1	1	1	2	0	0.5	0	0	0	0.5
**IQR**	0.75-3.5	0.75-1.0	0-2	0-2.25	0-1	0-1	0.0.25	0-1	0	0-2
	p = 0.13		p = 0.57		p = 0.53		p = 0.81		p = 0.063	

Participant	6 Six small cubes		7 Six pieces of clothes		8, 9, 10 Seven metallic circular disks, Six small balls, eight pins		Total	
	OH	NW	OH	NW	OH	NW	OH	NW
1	0	0	0	0	0	0	0	0
2	0	0	0	0	0	0	7	1
3	0	0	0	0	0	0	3	7
4	0	0	0	0	0	0	2	1
5	0	1	0	0	0	0	3	5
6	0	2	1	1	0	0	4	13
7	0	1	0	0	0	0	0	7
8	0	0	0	1	0	0	12	5
9	0	6	0	1	0	0	6	16
10	0	0	0	0	0	0	2	4
**Median**	0	0	0	0.3	0	0	3	5
**IQR**	0	0-1.25	0.1	0-1	0	0	1.5-6.25	1-8.5
	p = 0.13		p = 0.50		p = 1.00	p = 1.00	p = 0.26	

OH: Ottobock hand, NW: Our novel hand, IQR: Interquartile ranges.

Supplementary Table S3. Borg Scale Change.

**Participant**	**Borg Scale Change**	
	OH	NW
1	1	0
2	1	0
3	1	0
4	1	1
5	1	2
6	3	1
7	5	2.5
8	1	0
9	4.5	2
10	2	3
**Median**	1	1
**IQR**	1-1.375	0-2.125
	※p = 0.045	

OH: Ottobock hand, NW: Our novel hand, IQR: Interquartile ranges.

Supplementary Movie

The movie shows the functions of pinching, grasping, and gripping. In addition, video clips from a participant performing the 5th STEF (moving six wooden disks) are shown.
